# Editorial: Public Participation in Health Care: Exploring the Co-production of Knowledge

**DOI:** 10.3389/fsoc.2019.00073

**Published:** 2019-11-12

**Authors:** Gill Green, Annette Boaz, Maria Stuttaford

**Affiliations:** ^1^School of Health and Social Care, University of Essex, Colchester, United Kingdom; ^2^Centre for Applied Health and Social Care Research, Kingston University, Kingston upon Thames, United Kingdom

**Keywords:** participation, public involvement, health care, knowledge co-production, user voice

## User-Reference Group: David Bousfield, Lizzie Lloyd-Dehler, Sarah Rae

Participation and involvement of publics in the provision of health care is gaining traction as people are encouraged to become “discerning consumers” in seeking care and wellness from an increasingly diverse range of providers. In order to meet the demands of consumers, health care providers seek feedback from service users. It can be argued that the neoliberal agenda has appropriated participation and more recently there has been a discernible shift in the narrative of Patient & Public Involvement (PPI) and a change in terminology. There is now a greater emphasis on patients and the public having a stronger voice in order to share decision-making to co-produce research, services, and policy. However, this emancipatory potential of knowledge created through co-production does not fit easily with the continued neoliberal climate whereby health care provision is increasingly dictated by market forces. This collection of papers offers a global and provocative perspective on the tension between participation as emancipatory and reformative on the one hand and participation as a servant to neoliberal capital forces on the other.

Participation in healthcare is not a new concept and reflects a long history of political and structural struggle. Nevertheless, much of the recent literature on participation has been descriptive and evaluative. We therefore deliberately sought papers for this collection that offered a fresh and challenging perspective. Written by leading figures in the field of public participation, as well as some newer voices, the papers offer penetrating critiques of participation, making this an authoritative addition to the field. Papers include conceptual radical critiques as well as insightful commentaries drawing on experiences of those trying to implement new forms of working to break down traditional hierarchies.

A further distinct feature of this collection is the contribution of service users both as authors offering powerful user-led perspectives (e.g., Beresford; Goldsmith et al.; Rose and Kalathil), and as part of the editorial process. Frontiers provided financial support for the formation of a user-reference group of three people with diverse backgrounds to support the Editors. Their role was to screen papers for accessibility, interest and quality to make the collection more accessible and more relevant to the public. Authors were asked to provide a Plain English Summary and these were reviewed and revised in an iterative process with authors responding to feedback from the user-reference group. The Plain English Summaries are published here, following the editorial, as part of the collection. It is worth noting, however, that despite the willingness of the journal, the Editors and the user-reference group, this process was not straightforward and was time-consuming to implement. Academic publishing systems are not set up to include lay summaries and just as many of the articles in the collection demonstrate, it is challenging to add user input onto a pre-existing infrastructure. Based on our experience, we would suggest publishers consider making a lay summary a mandatory requirement and incorporating this into on-line submission systems.

The individual papers offer theoretical critique and empirical evidence about the potential for participation and co-production. The conceptual papers from Beresford, Madden and Speed, Paylor and McKevitt, Rose and Kalathil, and Stuttaford et al., all take a skeptical stance and provide critical perspectives about why, without significant changes, the adoption of the term co-production on its own will not lead to significant changes. Whether taking a historical (Beresford; Rose and Kalathil) or rights-based approach (Stuttaford et al.) or providing a policy (Madden and Speed) or sociological critique (Paylor and McKevitt) all authors broadly concur that while the policy narrative is supportive of participation, in reality structures and resources do not facilitate effective involvement. All caution that merely co-opting the term co-production within existing structures and processes is unlikely to lead to meaningful participation or transformational results. As a result the opportunity for public involvement to make a real difference gets lost and as Madden and Speed suggest doing good public involvement is like chasing a unicorn, a mythical creature that everyone talks about but has never actually been seen. To move forward, authors suggest that we need to re-engage with participatory traditions (Paylor and McKevitt) and focus on participation as a set of values and rights to strengthen participation (Stuttaford et al.). Writing from the field of mental health research, Beresford recommends a stronger funding base for user led organizations so they can drive innovation in involvement. Rose and Kalathil, while committed to user-led research, nevertheless caution that the user movement itself is not immune to power differentials and exclusion of marginalized, particularly “non-white” voices.

The theoretical/conceptual papers focusing on the shortcomings of PPI and skepticism about the ability to achieve co-production are complemented by a literature review and empirical contributions about the realities of co-production in practice. These provide detailed examples about why it is so challenging within the current structures to achieve spaces in which power differentials between professionals and publics can be overcome. Three papers, Green and Johns, O'Shea et al., and Goldsmith et al., focus on power between professionals and publics within decision-making processes. O'Shea et al. examine power in decision-making in clinical commissioning groups and identify a hierarchy of power, in which some professionals and public members are afforded more scope for influencing healthcare service development than others. A power differential is also evident in Green and Johns analysis of interviews with researchers and public partners, which they relate to the positivist framework which tends to dominate in health research as this privileges scientific over experiential knowledge. However, Goldsmith et al., show how knowledge from lived experience of mental health problems can be used in key decision-making even within the ultra-positivist framework of a randomized controlled trial. Co-production can be combined with randomized controlled trial methodology by incorporating service user perspectives throughout the research process. Whilst Goldsmith et al. offer a rare example of how co-production can work, the literature review by Tembo et al. show that in the early stages of research commissioning, consultation (which is frequently tokenistic) rather than co-creation of knowledge, decisions or processes, is the norm. And Twine et al. draw upon their experience of conducting longitudinal health research in an under-developed rural area of South Africa to challenge the idea of considering research participants as an experimental public separated from a notion of community and society. This serves as a timely reminder that research itself as well as public involvement *per se* should always be grounded in the community from whence it came to generate co-production and meaningful engagement.

The final three papers examine novel approaches to try to overcome some of the power differentials that have thus far predominated in public involvement. Zwama et al. report on an initiative in South Africa which applied a human rights-based approach to training health professionals about how to engage with lay representatives on health committees, a key mechanism for participation. Matthews and Papoulias describe the Exchange Network, which aims to redress power imbalance by creating a space for professionals and the public to make decisions on an equal basis. Suspending the rigidity of roles generally attributed to researchers and public partners can be transformative and is a necessary step toward co-production. Hundt et al. also suggest that innovative ways to engage stakeholders, such as use of research-based theater to perform research findings, can facilitate the co-production of knowledge. Post show discussions were found to transcend boundaries between the audience, actors and panel members to co-produce new knowledge.

Taken as a whole, these articles show that while civil engagement is more important than ever before, there are huge challenges. This collection provides theoretical insight and empirical evidence about why shared decision-making and co-production is so difficult to achieve. This builds on the limited evidence to support co-production's potential for transforming relationships between researchers/policymakers/practitioners and publics. While the terminology may have changed the experiential knowledge of service users is rarely afforded equal value to that of scientific/expert knowledge. We hope that this collection provides a theoretical and practical steer to address these challenges in order to achieve the co-production of knowledge so that experiential and professional knowledge is afforded equal authenticity.

## Author Contributions

All authors listed have made a substantial, direct and intellectual contribution to the work, and approved it for publication.

### Conflict of Interest

The authors declare that the research was conducted in the absence of any commercial or financial relationships that could be construed as a potential conflict of interest.

